# Fermented Dairy Products as Modulators of the Gut Microbiome: Greek Yogurt as a Model System

**DOI:** 10.1002/fsn3.71872

**Published:** 2026-05-11

**Authors:** Jason Dichter

**Affiliations:** ^1^ Department of Biological Sciences University of Pittsburgh Pittsburgh Pennsylvania USA

**Keywords:** fermented foods, greek yogurt, gut microbiota, metabolic health, probiotics, short‐chain fatty acids

## Abstract

Greek yogurt, characterized by its thick texture and higher protein content, contains reduced lactose levels while still preserving large colonies of active bacteria when compared to conventional (or “traditional”) yogurt. The starter cultures listed on its label do more than just ferment milk; they actively reshape the gut microbiome and adjust host physiology. This review examines currently available observations about distinct bacterial types within this product, especially regarding its effects on intestinal balance. The ability to produce short‐chain fatty acids is a property linked to these strains, along with the potential to stabilize gut lining function, adjust immunity patterns, aid blood sugar regulation, and even offer possible cardiovascular benefits. Greek yogurt's properties and potential differ from other fermented food items. Findings from experimental and clinical research suggest the lactic acid and Bifidobacterium species found in Greek yogurt contribute to increased microbiota variety, encourage growth of butyrate‐producing bacteria, and strengthen the intestinal lining. Inflammation levels are reduced by these microbes, leading to greater lactose tolerance, smoother digestion, and balanced metabolic activity. Still, much of the available data is limited because most studies do not distinguish Greek yogurt from conventional yogurt in their analyses, even though differences exist in live cultures, survival through digestion, and manufacturing. Fermented vegetables may offer wider microbe variety; however, consistency in bacterial strains and stronger clinical evidence gives Greek yogurt significance in nutritional and microbiome research. Future investigations should focus on Greek yogurt and prioritize direct comparisons to other fermented foods. To credibly refine dietary recommendations, improved microbial methodologies and expanded trials among diverse populations are warranted.

## Graphical abstract

Greek yogurt containing five defined bacterial strains ( *L. bulgaricus* , *S. thermophilus* , *L. acidophilus* , *B. lactis* , and *L. rhamnosus* ) delivers live cultures to the intestinal epithelium, where they interact with tight junctions, mucus, and villi. These microbes produce short‐chain fatty acids (SCFAs), acetate, propionate, and butyrate that contribute to seven key host health outcomes: barrier integrity, immune balance, glycemic control, reduced inflammation, lactose tolerance, cardiovascular protection, and metabolic health. Compared to wild‐fermented foods such as kefir, kimchi, sauerkraut, and tempeh, Greek yogurt offers defined cultures, reproducible composition, and stronger clinical evidence.

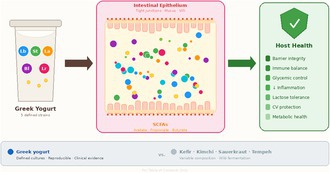

## 1 Introduction

With its rich content of live probiotics, Greek yogurt supports key aspects of gut wellness. Beneficial microbes present in Greek yogurt, for example, Lactobacillus and Bifidobacterium help sustain microbial variety within the intestines, along with reinforcing the lining of the digestive tract (Statista 2025). Eating yogurt often links to higher levels of helpful bacteria, stronger gut defenses, and smoother digestion (United States Department of Agriculture Economic Research Service 2025). Consistent intake of fermented foods has been associated with reduced systemic inflammation and favorable shifts in immune activity (Wastyk et al. 2021), although this effect has not yet been evaluated with Greek yogurt individually.

Every year, people in the United States eat an average of 13.8 pounds of yogurt, making it a common part of daily diets, and in 2022 alone, sales went beyond $7.2 billion (Statista 2025). About half, 51%, of that total belongs to Greek‐style products (United States Department of Agriculture Economic Research Service 2025), showing how much consumers favor this version. Yet even though it dominates shelves, scientific studies rarely separate Greek yogurt from other types, creating gaps in understanding. Because this yogurt variety packs more protein, less lactose, and hosts unique microbes, examining its isolated impact matters, especially regarding gut flora balance, immunity, and metabolism.

For the purpose of this review, Greek yogurt is defined as yogurt produced by fermentation with *Lactobacillus delbrueckii* subsp. bulgaricus and *Streptococcus thermophilus* , followed by a straining or mechanical separation step that removes whey (U.S. Food and Drug Administration 2025). This process yields a product with higher protein content (typically ≥ 10 g per serving), reduced lactose, and increased concentrations of lactic acid bacteria relative to unstrained conventional yogurt. Products marketed as “Greek‐style” that achieve thickness through added thickeners (e.g., modified starch, pectin, or milk protein concentrate) rather than straining are not considered the same and are excluded from this definition.

While several recent reviews have examined fermented foods and gut microbiota broadly (Leeuwendaal et al. 2022; Gao et al. 2025), none have specifically used Greek yogurt as a model system to evaluate the strength of evidence across different fermented dairy categories. This review advances the field by systematically distinguishing evidence derived from Greek yogurt, conventional yogurt, and isolated probiotic strains, revealing a critical gap: despite Greek yogurt's commercial dominance and distinct compositional profile, almost no randomized controlled trials (RCTs) have examined its specific effects on gut microbiome composition.

Focusing on how microbes in Greek yogurt operate, this analysis highlights short‐chain fatty acid generation, maintenance of intestinal lining, and control of immune responses. Beginning with a synthesis of available data, this review gathers insights from mechanistic studies, preclinical models, and human trials exploring how Greek yogurt influences microbial communities in the digestive tract. Sources include original research, comprehensive reviews, and meta‐analyses through the year 2025. Information was collected via academic databases such as PubMed and Google Scholar through search terms including Greek yogurt, fermented dairy, gut microbiome, probiotics, and short‐chain fatty acids.

### 1.1 Microbial Cultures in Greek Yogurt

Traditional Greek yogurt is produced using two primary starter cultures, *Lactobacillus delbrueckii* subsp. bulgaricus and *Streptococcus thermophilus* , with many commercial products also including supplemental probiotic strains such as *Lactobacillus acidophilus* and *Bifidobacterium animalis* subsp. lactis. Each strain acts uniquely in enduring stomach acid and producing diverse bioactive compounds. The detailed microbial composition of Greek yogurt, including starter culture interactions, added probiotic and postbiotic strains, and factors distinguishing Greek yogurt from conventional yogurt, is discussed in Section 3.

## 2 Fermented Foods and the Gut Microbiome

### 2.1 Microbial Diversity and Fermented Foods

Over thousands of years, food fermentation has been a significant portion of diets as it extends shelf life while enriching taste. Alongside preservation comes an array of active microbes and compounds formed during fermentation, some with potential health benefits (Dimidi et al. 2019). Lately, science has turned attention toward how these products influence microbial balance within the digestive system. Among them, yogurt stands alongside other fermented foods like kefir, which host unique blends of fungi and bacteria. Kimchi, another fermented food, appears in many meals, its tangy profile shaped by natural cultures thriving without heat treatment. Similarly, sauerkraut develops complexity through slow bacterial activity. Tempeh emerges from mold‐assisted transformation of soybeans, introducing yet another pattern of organisms. Even sourdough bread made with wild starters carries traces of diverse bacterial species (Frias et al. 2017; Kaur et al. 2022). This diversity matters because findings from one fermented food cannot be assumed to apply to another, yet nutritional guidelines and clinical studies routinely treat them as the same (Marco et al. 2017).

Fewer microbes survive industrial handling than one might expect, with viable counts often declining by several log orders during processing and storage (Mjaaseth and Lefevre 2020), yet many still arrive intact through digestion into the intestines. Important, since these live cultures come not in isolated supplements but embedded naturally in fermented items, along with biochemical byproducts formed during preparation. The surrounding food structure seems to aid survival, colonization, and helpful functions once inside the body (Rezac et al. 2018).

Evidence continues to grow showing fermented foods affect gut bacteria in measurable ways. One major study found people eating more of these foods had greater variety in their gut microbes along with lower markers of systemic inflammation (Afzaal et al. 2022). Though most introduced microbes pass through without staying permanently, their short stay still shifts native communities, limits harmful strains, and encourages organisms like *Akkermansia* and *Faecalibacterium* to thrive (Kaur et al. 2022; Choi et al. 2025).

### 2.2 Metabolites From Fermentation

Beyond live microbes, fermented items affect wellness through various means. During fermentation, substances like lactic acid, short‐chain fatty acids (SCFAs), which consist primarily of acetate, propionate, and butyrate, which are produced when gut bacteria ferment digested food, along with peptides, vitamins, and antioxidants form. Lactic acid presence causes a lower pH, which directly opposes harmful microbes while supporting beneficial ones. Acetate or propionate, among other fermentation byproducts, aid the lining of the intestine, adjust body defenses, and play roles in metabolic processes involving glucose and lipids (Settachaimongkon et al. 2014; Ayivi et al. 2020).

What sets fermented foods apart lies in the combination of live microbes and the compounds they produce during fermentation. Still, it is important to understand the differences across types when it comes to microbial makeup or chemical output, as different starting materials and utilized microbes cause different outcomes. For example, kefir hosts more diverse organisms than yogurt does, and in contrast, kimchi and tempeh introduce unique blends of microbes and substances formed through their process (Mchiouer et al. 2017). Because these variations exist, drawing broad conclusions about health effects becomes problematic. To understand how each food affects well‐being, attention to individual species, ingredients used, and methods applied matters substantially. These variations across fermented foods make it necessary to examine each product in its own terms, yet numerous studies still treat Greek yogurt like any other variety, despite significant differences in composition and production.

## 3 Microbial Composition of Greek Yogurt

Following fermentation with specific bacteria, milk becomes Greek yogurt, a substance high in protein and stable in microbial composition. During this transformation, the yogurt thickens while also maintaining uniformity in microbe types across batches. Because its biological makeup remains predictable, it serves as a great model for studying how dietary microbes interact with the human body. Beginning with foundational cultures needed for fermentation, many samples contain additional live organisms as some brands include added probiotics, not by accident but to conjure additional health benefits.

### 3.1 Starter Cultures in Greek Yogurt

The main microbes behind Greek yogurt fermentation include *Lactobacillus delbrueckii* subsp. *bulgaricus* and *Streptococcus thermophilus* . Starting quickly, *S. thermophilus* breaks down lactose, releasing formate and carbon dioxide, and these substances support *L. bulgaricus* development. As things progress, *L. bulgaricus* splits milk proteins into amino acids and peptides, which feed *S. thermophilus* metabolism (Hill et al. 2014; Southcott et al. 2008). Because of this interdependence, Greek yogurt is imparted with its characteristic acidity and texture during fermentation. Afterward, the yogurt is strained, removing most of the whey, which leaves a denser mix rich in protein and bacterial byproducts, their concentrations defining the final composition.

### 3.2 Added Probiotic and Postbiotic Strains

Beyond the common starter bacteria, some store‐bought Greek yogurts include extra microbial types, with *Lactobacillus acidophilus* , *Lacticaseibacillus rhamnosus*, and *Bifidobacterium animalis* subsp. *lactis* among them. These microbes are selected for their resilience in the digestive environment, and they stick well to gut linings while making helpful compounds like SCFAs. *L. acidophilus* forms lactate and acetate from breaking down sugars, with these substances tied to stronger intestinal walls. *B. lactis* improves the digestion of lactose, supports the immune system, and acetate release encourages growth of neighboring beneficial bacteria (Mjaaseth and Lefevre 2020; Gao et al. 2025). After antibiotics disturb balance, *L. rhamnosus* often stands out by helping restore protective lining in the gut (Escalona‐Jiménez et al. 2022; Markowiak‐Kopeć and Śliżewska 2020; Linninge et al. 2019).

Survival of bacterial strains in Greek yogurt depends on pH, temperature during culturing, storage conditions after production, and shelf life. Research shows fewer active microbes remain as the item nears its best‐by date (Chandrasekaran et al. 2024); even so, functioning and non‐functioning microbes, together with their cellular components, known as postbiotics (i.e., bioactive compounds released by or produced through the metabolic activity of microorganisms, including cell wall fragments, metabolites, and extracellular polysaccharides), may support digestive function and immunity (Khuropakhonphong et al. 2021). Because of this, eating Greek yogurt introduces more than just living cultures, it includes various metabolite products and cell fragments, each playing a role in potential health benefits.

### 3.3 Distinguishing Greek Yogurt

**FIGURE 1:**
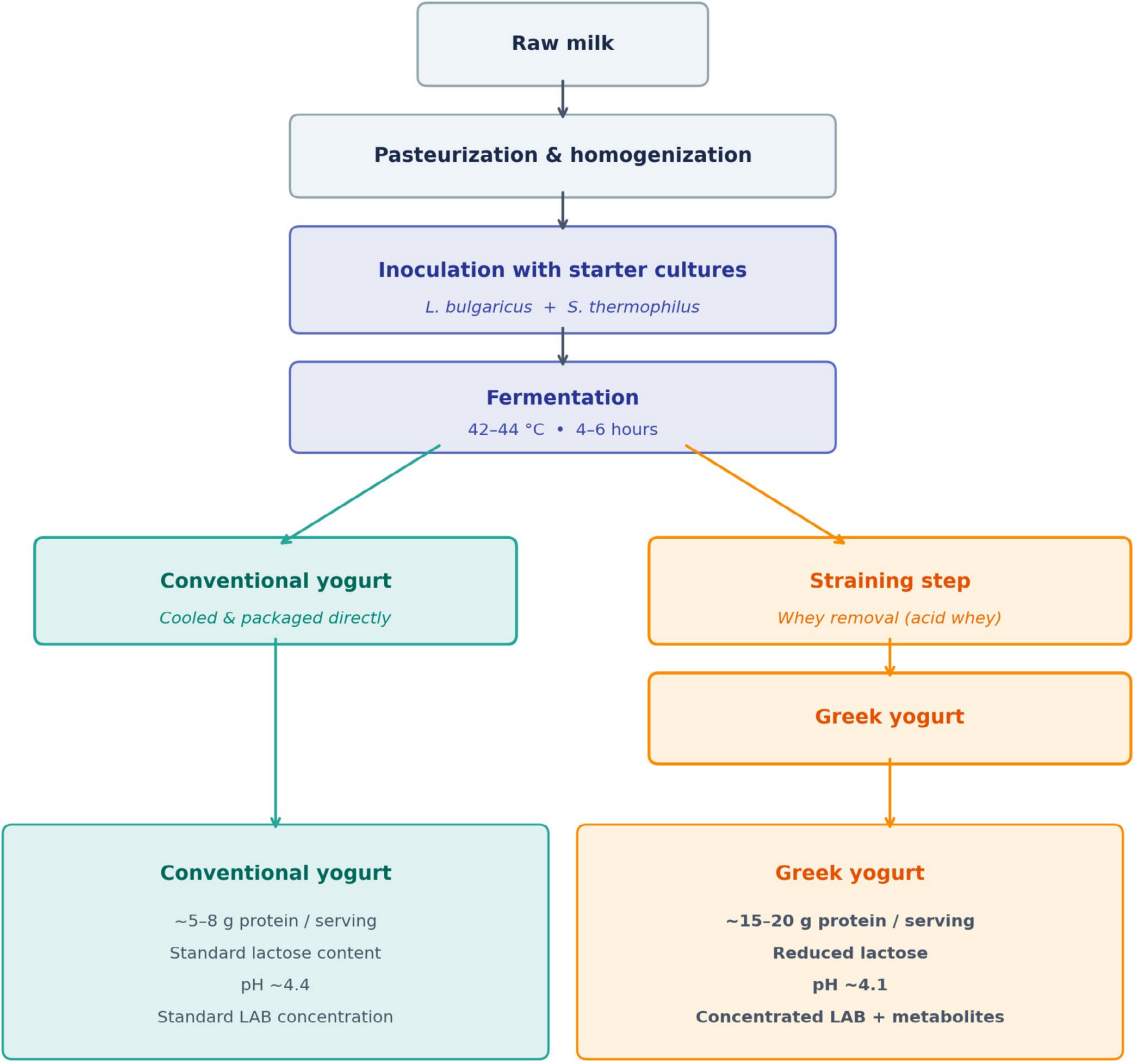
Schematic comparison of Greek yogurt and conventional yogurt production processes. Both products share initial steps of pasteurization, homogenization, inoculation with starter cultures ( *L. delbrueckii* subsp. *bulgaricus* and *S. thermophilus* ), and fermentation. The key divergence occurs at the straining step, where Greek yogurt undergoes whey removal, resulting in higher protein content (~15–20 g per serving vs. ~5–8 g), reduced lactose levels, increased acidity, and higher concentrations of lactic acid bacteria (LAB) and their metabolic byproducts.

Greek yogurt distinguishes itself from regular yogurt primarily through its unique production process (Figure 1). The straining technique significantly increases protein content and reduces lactose levels, which in turn influences the microbial composition present in the final product. This process changes the fermentation process, selectively promoting the growth of specific bacterial populations. Research indicates that Greek yogurt typically contains higher concentrations of lactic acid bacteria (LAB), and products supplemented with probiotics can achieve even greater microbial counts. In addition, because most Greek yogurts undergo limited post‐fermentation heat treatment, a greater proportion of these beneficial microbes remain alive and utilizable compared to more heavily processed or flavored yogurts, which often experience substantial microbial loss. The higher acidity and solid content characteristic of Greek yogurt further support the survival and stability of these bacteria over time (Xiong et al. 2022).

In summary, Greek yogurt represents an intricately balanced microbial ecosystem, comprising cooperative starter cultures, selectively added probiotic strains, and a spectrum of metabolic byproducts. The interactions among these components support Greek yogurt's effects on gastrointestinal function, immune regulation, and overall metabolic health (Table 1).

**TABLE 1:** Bacterial strains present in Greek yogurt, their functional attributes, and health‐related effects.

Strain	Category	Key functional traits	Primary health‐related effects	Representative sources
*Lactobacillus delbrueckii* subsp. *bulgaricus*	Starter culture	Strong lactose fermentation; high lactic acid production; proteolytic activity	Lowers gut pH, supports beneficial microbes, contributes to gut barrier support	(Mchiouer et al. 2017; Hill et al. 2014)
*Streptococcus thermophilus*	Starter culture	Rapid lactose metabolism; produces formate & CO_2_; synergistic growth with *L. bulgaricus*	Enhances growth of other LAB; improves lactose digestion	(Mchiouer et al. 2017; Khuropakhonphong et al. 2021)
*Lactobacillus acidophilus*	Added probiotic	Acid‐ and bile‐tolerant; adheres to intestinal epithelium; SCFA producer	Supports digestive health, improves gut barrier, may reduce GI discomfort	(Gao et al. 2025; Linninge et al. 2019)
*Bifidobacterium animalis* subsp. *lactis*	Added probiotic	Acetate producer; improves colonic fermentation; survives dairy matrix well	Enhances lactose digestion, supports immune modulation, enriches beneficial gut taxa	(Mjaaseth and Lefevre 2020; Gao et al. 2025)
*Lacticaseibacillus rhamnosus*	Added probiotic	Strong epithelial adhesion; immunomodulatory; known for resilience after dysbiosis	Improves gut barrier integrity, helps restore microbiota after disruption	(Southcott et al. 2008; Escalona‐Jiménez et al. 2022)
Heat‐killed and non‐viable LAB (postbiotics)	Naturally present during/after fermentation	Cell wall fragments, metabolites survive storage; immune‐active components	Reduces inflammation, enhances barrier even without live bacterial colonization	(Markowiak‐Kopeć and Śliżewska 2020; Linninge et al. 2019)

## 4 Mechanisms of Health Benefits

**FIGURE 2:**
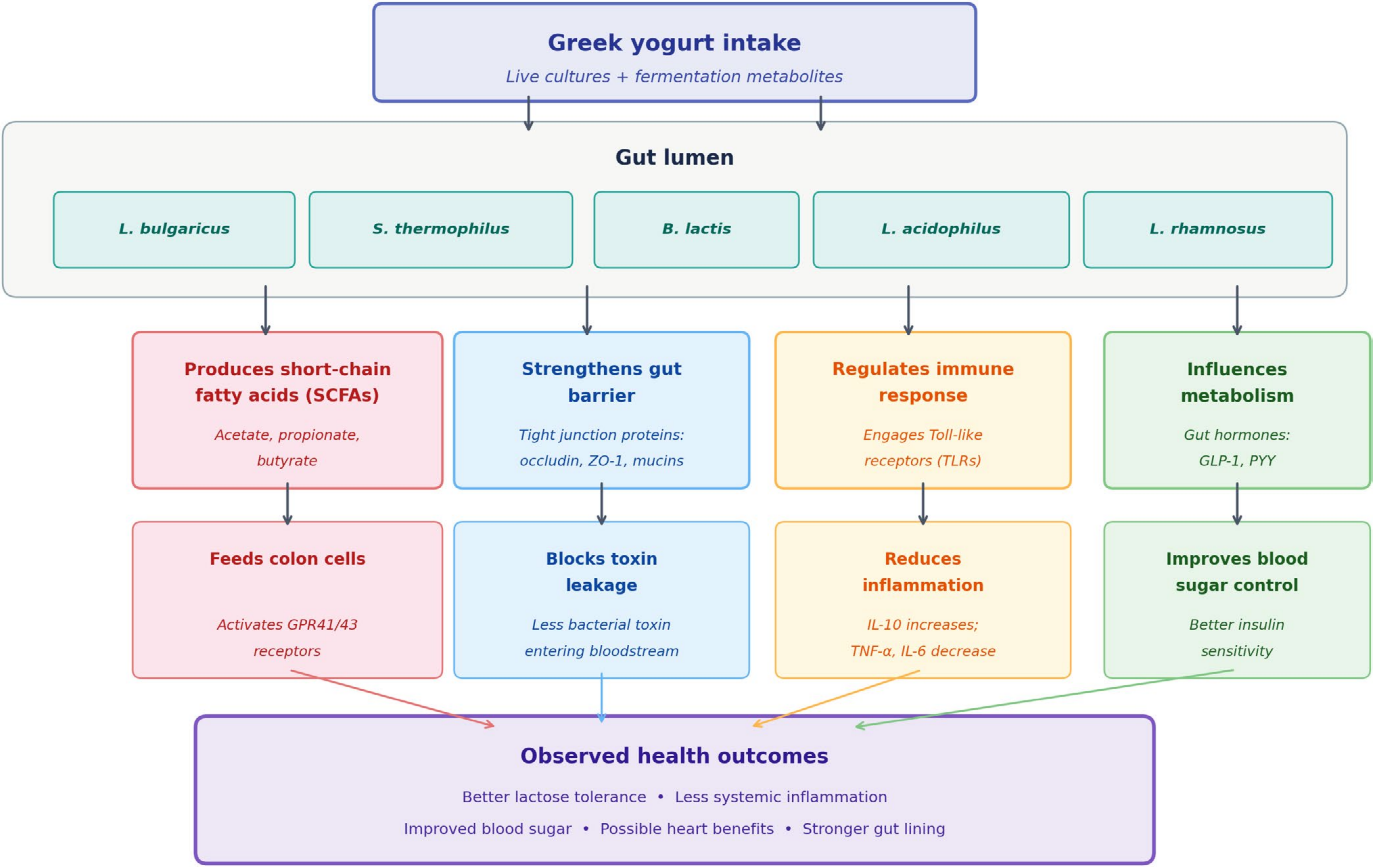
Mechanistic diagram illustrating how Greek yogurt‐derived microbes and metabolites influence host health through four interconnected pathways. Probiotic strains ( *L. bulgaricus* , *S. thermophilus* , *B. animalis* subsp. *lactis*, *L. acidophilus* ) transit the gut lumen and exert effects through: (1) Enhanced short‐chain fatty acid (SCFA) production, providing colonocyte fuel and activating GPR41/GPR43 receptors; (2) Reinforcement of the intestinal barrier via upregulation of tight junction proteins (occludin, ZO‐1) and mucin secretion, reducing lipopolysaccharide (LPS) translocation; (3) Modulation of immune function through Toll‐like receptor engagement, promoting anti‐inflammatory cytokines (IL‐10) while suppressing pro‐inflammatory mediators (TNF‐α, IL‐6); and (4) Metabolic regulation via gut hormone signaling (GLP‐1, PYY) and bile acid metabolism. These pathways converge on clinical outcomes including improved lactose tolerance, reduced systemic inflammation, glycemic control, and potential cardiovascular benefits. Evidence strength varies by outcome domain (see Section 5).

Greek yogurt's health benefits are associated with four interconnected mechanisms (Figure 2), each driven by the specific microbial profile discussed above. The ability to examine these pathways individually is thanks to Greek yogurt's standardized composition, something not easily achieved in other fermented foods. The probiotic strains commonly found in Greek yogurt, such as those from the *Lactobacillus* and *Bifidobacterium* species, generally do not establish long‐term residence in the gut. Nevertheless, their transit through the digestive tract exerts notable physiological effects, including enhanced production of SCFAs, reinforcement of the intestinal barrier, regulation of the immune response, and adjustment of metabolic processes. Over time, research across populations and controlled clinical studies have shown consistent yogurt consumption connects closely with improved gut health, reduced systemic inflammation, and favorable metabolic outcomes.

### 4.1 Enhancement of Short‐Chain Fatty Acid Production

Boosting levels of SCFAs, especially acetate, propionate, and butyrate, forms a core reason behind Greek yogurt's influence on digestive well‐being. Though often overlooked, LAB and bifidobacteria do more than generate SCFAs themselves; they assist native microbes like *Faecalibacterium* species in producing butyrate efficiently. Findings by Markowiak‐Kopeć and Śliżewska (2020), based on studies of isolated Lactobacillus and Bifidobacterium strains rather than Greek yogurt, show certain *Lactobacillus* and *Bifidobacterium* types encourage growth among butyrate‐producing bacteria, raising total SCFA output while reducing colon acidity. With pH dropping, harmful microbes struggle to survive, whereas beneficial ones gain stability within the intestinal environment.

Beyond the gut, SCFAs impact multiple body systems. Upon absorption, acetate and propionate affect liver activity, adipose cells, and immune regulation. Butyrate, instead, fuels colon lining cells while aiding integrity and recovery of the gut wall. These actions occur largely through specific G protein‐coupled cell receptors, GPR41 and GPR43, which regulate inflammation, insulin sensitivity, and release of digestive hormone signals. Evidence further supports these mechanisms: adults consuming Bulgarian yogurt, an unstrained yogurt variety, showed higher stool levels of SCFAs, specifically acetate, in adult women as noted by Khuropakhonphong et al. (2021). From such data emerges a consistent picture, one where microbes from fermented dairy alter fermentation outputs in the bowel, which results in detectable bodily changes. Notably, one of the few studies using Greek yogurt specifically found that 42 days of daily plain Greek yogurt consumption led to increased abundance of *L. casei* in the gut, a well‐documented probiotic species associated with anti‐hypertensive, antioxidant, and immune‐regulatory effects (Pimentel et al. 2021), though overall community diversity changes were not statistically significant, likely due to the small sample size (Lisko et al. 2017).

### 4.2 Reinforcement of the Intestinal Barrier

Among dairy options, Greek yogurt stands apart due to its influence on digestive resilience. Because of its composition, it supports the lining of the intestines in ways that go beyond satiety. The internal barrier helps block unwanted substances, like toxins and bacterial fragments including lipopolysaccharide (LPS) from entering the bloodstream. When compromised, which some refer to as “leaky gut,” the body may respond with heightened immune activity and imbalance in metabolism. Such weakening often coincides with gastrointestinal discomfort.

Protection offered by Greek yogurt mainly comes from the live cultures it contains. Studies show certain helpful bacteria, like lactobacilli and bifidobacterial, increase levels of key cell‐binding proteins such as occludin, claudin‐1, and ZO‐1 (Guo et al. 2025). Because of this shift, gut lining integrity strengthens over time. Along similar lines, these microbes cause a higher output of mucins, thickening the inner mucus shield. As a result, the movement of harmful substances like LPS slows down and irritation in intestinal tissues decreases. Findings seen in lab animals align closely; for example, Southcott et al. (2008), using an unspecified probiotic yogurt, demonstrated that probiotic yogurt supplementation preserves gut barrier function and reduces inflammation‐induced tissue damage in stress‐exposed rats.

Fundamental is the idea that elements within Greek yogurt, probiotics among them, help maintain the lining of the digestive tract. With regular intake, internal defenses adjust, changing how inflammation arises when barriers weaken, resulting in a measurable effect on barrier integrity.

### 4.3 Regulation of Immune Function and Attenuation of Inflammation

Beyond localized gastrointestinal effects, Greek yogurt may affect systemic immune responses. Research led by Plaza‐Díaz et al. (2019), examining probiotic mechanisms broadly rather than Greek yogurt specifically, reveals that probiotic components engage immune sensors like Toll‐like receptors (TLRs). Because of this engagement, signals that drive inflammation, such as those from nuclear factor NF‐κB, are less active. As a result, the body leans into anti‐inflammatory immune activity, marked by higher levels of regulatory cytokines like interleukin‐10 (IL‐10). At the same time, molecules linked to swelling, including tumor necrosis factor‐alpha (TNF‐α) and interleukin‐6 (IL‐6), decrease in presence.

Beyond this, gut microbes produce SCFAs when exposed to components in yogurt, adding to the overall effect. One such compound, butyrate, supports growth of regulatory T cells while limiting gene activity tied to inflammatory processes. As noted by Xiong et al. (2022) these metabolites influence how immune and epithelial cells behave, revealing their wide‐ranging role in reducing inflammation. With stronger gut lining integrity alongside balanced immune responses, the link between daily yogurt consumption and lower systemic inflammation becomes more visible. Supporting this, a recent randomized clinical trial (RCT) using Greek yogurt specifically found that 12 weeks of post‐exercise Greek yogurt consumption modulated markers of systemic inflammation in healthy young males compared to an equal calorie carbohydrate control (Fraschetti et al. 2025).

### 4.4 Metabolic Health and Host Physiology

Beginning with digestion, Greek yogurt supports metabolic function. Through fermentation activity, it encourages formation of SCFAs. These compounds help maintain intestinal lining integrity while influencing gut‐derived hormone signaling. From within the colon, SCFAs prompt release of certain peptides, including glucagon‐like peptide‐1 (GLP‐1) and peptide YY (PYY), involved in glucose control. Such substances slow stomach outflow, improve insulin responsiveness, and aid steady blood sugar levels. As shown in work by Gao et al. (2025), fermented milk variants broadly (not Greek yogurt specifically) alter bile acid metabolism, leading to shifts in lipid absorption, cholesterol management, and energy expenditure.

Research findings align with results seen in controlled trials. One study by Bui and Marco (2025), examining fermented dairy products broadly rather than Greek yogurt in isolation, shows a link between higher consumption of fermented dairy products and better digestive function, lower levels of inflammation, and more balanced metabolism. What stands out is that such improvements occur even without major reductions in body weight, indicating that substances from yogurt, like live cultures and their byproducts, influence metabolic activity directly instead of acting only via shifts in size or body mass. Beyond metabolic markers, Greek yogurt has also demonstrated effects on bone health, as a 12‐week RCT in university‐aged males showed that Greek yogurt consumption during high‐impact exercise increased bone formation markers (procollagen type I N‐terminal propeptide; P1NP) compared to a placebo devoid of protein and calcium (Bridge et al. 2020).

## 5 Clinical Health Effects of Greek Yogurt Consumption

Clinical evidence linking yogurt to measurable health outcomes is growing but remains inconsistent. Certain areas, particularly lactose intolerance and blood sugar, depend on relatively strong trial data, while cardiovascular and oncological findings still rely heavily on observational studies with limited ability to differentiate Greek yogurt's specific contribution.

### 5.1 Metabolic Health and Type 2 Diabetes

Despite scattered results, most evidence shows benefits when fermented dairy products influence glycemic control. In one wide‐ranging review of clinical experiments, eating items like cultured‐milk yogurts (conventional and probiotic‐enhanced varieties) linked to lower levels of glycated hemoglobin (HbA1c), along with decreased fasting glucose and insulin readings in people showing prediabetes or already managing type 2 diabetes (Barengolts et al. 2019; Teo et al. 2024). When research focuses specifically on enhanced probiotic versions versus standard yogurt types in overweight or diabetic groups, results tend to show minimal differences (Barengolts et al. 2019). Shaping long‐term resistance against type 2 likely depends more on consistent food choices, including healthy fats, slow‐releasing carbs, and positive lifestyle habits (Ardisson Korat et al. 2014). Even so, certain brief improvements have been observed: in one of the few trials using Greek yogurt specifically, female participants eating Greek yogurt topped with granola clusters at breakfast showed post‐meal glucose values drop by around 52% relative to peers served fiber‐rich plant‐based cereal blends without dairy (Mather 2018).

### 5.2 Gastrointestinal Health and Lactose Intolerance

Yogurt's main benefits lie in its ability to support digestion, mainly by improving gut activity and alleviating related discomforts. Because it undergoes fermentation, lactose digestion is facilitated more effectively, which is most important for those who struggle with lactose intolerance. Helpful microbes inside remain active through the digestive system, breaking down lactose along the way instead of leaving it undigested. As a result, fewer unpleasant reactions occur after eating dairy products like these. Another noticeable shift is stomach contents move out slower after yogurt consumption, possibly further easing digestive discomfort (He et al. 2008; Savaiano 2014). This research examined conventional yogurt and bifidobacteria supplementation rather than Greek yogurt specifically. Studies point toward certain strains such as Bifidobacteria reshaping microbial balance in lactose intolerant individuals, cutting back on typical flare‐ups linked to intolerance. Symptom intensity drops when regular intake occurs, indicating internal adjustments take place over time (He et al. 2008).

Among dairy options, some derived from fermentation could bring certain benefits for people experiencing digestive issues. It appears that fermented dairy supports a broader variety of microbes in the intestine, easing discomfort like cramps or unstable bowel movements, especially noticeable in cases involving irritable bowel syndrome (IBS) or inflammatory bowel disease (IBD) (Chonnacháin et al. 2024). Such shifts often align with rising numbers of helpful organisms including Lactobacillus and Bifidobacterium, along with higher concentrations of SCFAs known to maintain intestinal balance. When it comes to managing IBD, introducing live microbial strains has been seen as advisable, with findings showing they help sustain inactive phases of ulcerative colitis and reduce the occurrence of pouchitis. Rather than stimulating harmful agents, these cultures tend to limit unwanted microbes, lower inflammatory biomarkers, and modulate immune function in the intestinal lining (Jones and Foxx‐Orenstein 2007).

### 5.3 Cardiovascular and Oncological Outcomes

Fermented dairy products tend to show stronger links to heart and cancer protection than unfermented versions. Studies based on conventional yogurt and fermented dairy broadly (rather than Greek yogurt specifically) suggest people who eat more low‐fat fermented items like yogurt face fewer cardiovascular issues. On the other hand, those consuming larger amounts of non‐fermented low‐fat dairy may experience a rise in cardiovascular risks (Koskinen et al. 2018; Aziz et al. 2024).

It appears that greater consumption of dairy links to less widespread inflammation within the body. Evidence suggests people who include more dairy in their diet tend to show diminished levels of markers tied to inflammation, among them C‐reactive protein (CRP), TNF‐α, and IL‐6, while also displaying increased amounts of adiponectin, a substance known to counteract inflammation (Moosavian et al. 2020; Luo et al. 2021). When it comes to cancer, findings from extensive long‐term research, including projects like the Nurses' Health Study and the Health Professionals Follow‐Up Study, indicate weekly intake of at least one portion of yogurt may relate to lower chances of developing proximal colon cancer; however, no clear connection has emerged concerning death rates from colorectal cancer overall (Michels et al. 2020).

### 5.4 Appetite Regulation and Satiety

One claim often made involves Greek yogurt leading to greater satiety because of its high protein content. In one of the few trials directly comparing Greek yogurt to conventional yogurt, healthy women were given either Greek yogurt with 14 g of protein or standard yogurt offering 5 g (Ortinau et al. 2013). Results showed no meaningful difference in how satisfied they felt afterward. In another instance, a fermented dairy snack rich in protein faced off against a plant‐based alternative high in fiber. Despite differing compositions, both managed to lower appetite and cut down eating at later meals across multiple hours post‐breakfast (Mather 2018). These findings suggest that satiety depends on more than just protein content alone, as bioactive compounds and microbial metabolites may contribute through pathways that macronutrient quantity does not influence.

### 5.5 Limitations and Conflicting Evidence

Despite the broadly favorable findings reviewed above, several important limitations warrant acknowledgment. First, the vast majority of studies cited in this review did not use Greek yogurt specifically as the intervention. As summarized in Table 2, most evidence derives from conventional yogurt, unspecified fermented dairy, or isolated probiotic strains studied outside a food matrix. To date, no large‐scale randomized controlled trials have examined Greek yogurt's effect on gut microbiome composition as a primary outcome. The few Greek yogurt‐specific RCTs that exist have focused on bone formation (Bridge et al. 2020), systemic inflammation during exercise (Fraschetti et al. 2025), appetite regulation (Ortinau et al. 2013), and postprandial glucose (Mather 2018), rather than on microbiome endpoints. One small study using plain Greek yogurt over 42 days found individual‐specific shifts in gut bacterial composition but no statistically significant changes in overall diversity, likely due to its limited sample size of five analyzed subjects (Lisko et al. 2017). This represents a critical gap: the central premise of this review, that Greek yogurt modulates the gut microbiome, rests largely on extrapolation from conventional yogurt and isolated strain studies.

**TABLE 2:** Summary of evidence sources cited in this review, classified by yogurt type or intervention used.

Study	Intervention type	Health outcome	Evidence category
Ortinau et al. 2013	Greek yogurt versus conventional yogurt	Appetite regulation, satiety	(a) Greek yogurt
Mather 2018	Greek yogurt with granola versus plant‐based cereal	Postprandial glucose, satiety	(a) Greek yogurt
Lisko et al. 2017	Plain Greek yogurt (0% fat), 42 days	GI microbiome composition and diversity	(a) Greek yogurt
Bridge et al. 2020	Greek yogurt versus placebo pudding, 12 weeks + exercise	Bone formation markers (P1NP)	(a) Greek yogurt
Fraschetti et al. 2025	Greek yogurt versus carbohydrate pudding, 12 weeks + exercise	Systemic inflammation markers	(a) Greek yogurt
Yogurt (type unspecified)	Gut microbial diversity, Akkermansia enrichment	(b) Conventional yogurt	
Khuropakhonphong et al. 2021	Bulgarian yogurt	Constipation relief, fecal SCFAs	(b) Conventional yogurt
Southcott et al. 2008	Probiotic yogurt (unspecified type)	Gut barrier function (rat model)	(b) Conventional yogurt
Barengolts et al. 2019	Probiotic yogurt (meta‐analysis of conventional types)	Glycemic control in T2D/obesity	(b) Conventional yogurt
He et al. 2008	Conventional yogurt + bifidobacteria supplement	Colonic microbiota in lactose intolerance	(b) Conventional yogurt
Wastyk et al. 2021	High‐fermented‐food diet (mixed fermented foods)	Immune status, microbiota diversity	(b) Fermented foods broadly
Koskinen et al. 2018	Fermented versus non‐fermented dairy (observational)	Coronary heart disease risk	(b) Fermented dairy broadly
Michels et al. 2020	Yogurt (type unspecified, observational cohort)	Colorectal cancer incidence	(b) Conventional yogurt
Markowiak‐Kopeć and Śliżewska 2020	Isolated Lactobacillus and Bifidobacterium strains	SCFA production	(c) Isolated probiotic strains
Plaza‐Díaz et al. 2019	Probiotic mechanisms (review of isolated strains)	Immune modulation, TLR signaling	(c) Isolated probiotic strains
Linninge et al. 2019	*L. rhamnosus* supplementation (isolated strain)	Post‐antibiotic microbiota recovery	(c) Isolated probiotic strains

*Note:* Evidence categories: (a) Greek yogurt specifically; (b) conventional yogurt, fermented dairy broadly, or yogurt type unspecified; (c) isolated probiotic strains studied outside a yogurt matrix. This distinction is critical because health effects observed with isolated strains or conventional yogurt cannot be assumed to apply directly to Greek yogurt without dedicated trials.

Second, this review has presented a largely positive view of Greek yogurt's health effects, but null and negative findings exist across several domains. Beyond the satiety null results reported by Ortinau et al. (2013), studies examining yogurt's effect on overall gut microbial diversity have yielded mixed results, with some reporting no significant changes (Lisko et al. 2017). Furthermore, the commercial Greek yogurt landscape introduces confounds not addressed in most research as many products contain substantial added sugars, artificial flavors, or undergo post‐fermentation heat treatment that reduces viable microbial counts. Potential adverse effects, including digestive discomfort in individuals with undiagnosed dairy sensitivities and the high calories of flavored varieties, have not been systematically studied. Publication bias likely favors positive outcomes, and the short duration of most trials (typically 4–12 weeks) limits conclusions about long‐term microbiome or metabolic effects.

Third, the definition of “Greek yogurt” itself lacks standardization. Commercial products vary widely in protein content (ranging from approximately 10 to 20 g per serving), straining methods (traditional cloth straining versus centrifugal separation versus thickener addition), fat content, and the presence or absence of supplemental probiotic strains. No regulatory standard defines “Greek yogurt” in the United States, and products marketed as “Greek‐style” may not share the same compositional profile. This heterogeneity makes cross‐study comparison difficult and underscores the need for future research to clearly report the specific product used, including starter cultures, added probiotics, protein and lactose content, and viable microbial counts at the time of consumption.

## 6 Yogurt Versus Other Fermented Foods

Live microbes along with their fermentation byproducts enter the digestive tract through certain foods, altering gut bacteria, shifting immune responses, and causing changes in metabolism. Still, which microbes appear, and what they do, depends heavily upon the type of product and the fermentation approach used (Suvarna and Boby 2005; Pihelgas et al. 2025). Population‐level data show people eating these items regularly carry unique patterns in both microbes and metabolite profiles when compared to non‐consumers. Such shifts involve specific microbes present plus natural substances tied directly to dietary habits (Cha et al. 2024). Of all such options available, yogurt, especially Greek yogurt, stands out as a notable example. Manufactured using specific microbial strains alongside uniform procedures, it delivers consistent products every time. Because results repeat across batches, researchers often turn to yogurt when exploring how food shapes intestinal microbes (Leeuwendaal et al. 2022; Taylor et al. 2020; Hadjimbei et al. 2022; Chonnacháin et al. 2024).

### 6.1 Product Standardization

Fermented dairy items like yogurt differ from numerous fermented foods due to their predictable makeup. Thanks to defined strains of lactic acid microbes, batch‐to‐batch similarity stays high. Spontaneously cultured alternatives lack such reliability, as their shifting ingredients and environments shape different microbiomes (Leeuwendaal et al. 2022; Taylor et al. 2020; Pihelgas et al. 2025). Clinical data consistently back the steady microbial profile found in these dairy‐based options, especially when enhanced with additional probiotic strains. What emerges is less fluctuation and more reproducibility. Frequent intake associates with better lactose breakdown, fewer digestive issues, while also showing positive impacts on heart and metabolism functions (Leeuwendaal et al. 2022; Taylor et al. 2020). Unlike those products, plant‐based ferments like kimchi rely on microbes already found on fresh produce or in air and soil, which leads to wide shifts in microbe types based on ingredient sources, salt levels, and fermentation length (Suvarna and Boby 2005; Kim et al. 2024). The mix of organisms forming during each vegetable batch is much different from dairy‐derived cultures. For clarity in microbiome research, separating these categories becomes necessary because their biological makeup and health influences are clearly dissimilar (Suvarna and Boby 2005; Tamang et al. 2016).

### 6.2 Fermented Vegetable Products

Fermented dairy items show clear links to digestive and metabolic health, supported by several studies (Leeuwendaal et al. 2022; Taylor et al. 2020; Pihelgas et al. 2025). With consistent consumption, yogurt and similar probiotic‐fortified options may ease lactose digestion and reduce inflammation. Symptom relief within the gastrointestinal tract appears occasionally, though outcomes differ across individuals. Yet such results depend strongly on the exact type of product used, making generalizations difficult (Leeuwendaal et al. 2022; Taylor et al. 2020).

Still, scientific evidence offers only modest support so far when it comes to fermented vegetables, even if newer work is slowly uncovering possible impacts. While signs point toward shifts in gut bacteria and subtle effects on body composition after consumption, many current investigations suffer from small participant groups and short durations (Tamang et al. 2016). In lab animals, items like kimchi have shifted both microbial balance and metabolic markers among those eating high‐fat diets, correlating with improvements in obesity‐related outcomes. Yet applying rodent results directly to people risks overstating what they truly mean (Kim et al. 2024). Kimchi preparations draw attention due to specific microbes showing beneficial traits under lab conditions; however, such foods fail to fulfill certain criteria under established probiotic standards, including the use of defined strains with demonstrated health benefits and evidence of survival through the gastrointestinal tract. Emphasis emerges on precise microbial profiling and clinical studies in people; only then can the health benefits of these fermented vegetables be measured (Cha et al. 2024).

### 6.3 Greek Yogurt as a Model System

A growing number of observational and clinical investigations point to links between eating fermented foods and greater variety in gut microbes, along with better markers of immunity and metabolism. Still, pinpointing specific contributions from single items within such diets is difficult because real‐world eating habits are rarely simple (Taylor et al. 2020; Afzaal et al. 2022; Suvarna and Boby 2005).

Research commonly selects Greek yogurt as a representative fermented food. Because its manufacturing relies on defined bacterial starters, sometimes enhanced with probiotics, the method delivers a uniform composition that consists of abundant protein and reduced lactose levels (Leeuwendaal et al. 2022; Pihelgas et al. 2025; Chonnacháin et al. 2024). Given this reproducibility, investigators achieve accurate links between targeted physiological effects and distinct microbes or their metabolites, and such clarity remains uncommon when examining variable preparations like kimchi or homemade sauerkraut (Pihelgas et al. 2025; Chonnacháin et al. 2024; Suvarna and Boby 2005).

Although kefir or fermented vegetables contain a broader spectrum of microbes and active compounds, Greek yogurt is better suited in research focused on specific strains or metabolites influencing gut flora, immunity, and metabolism (Leeuwendaal et al. 2022; Taylor et al. 2020; Pihelgas et al. 2025; Suvarna and Boby 2005).

## 7 Clinical Recommendations

Recent findings suggest Greek yogurt may aid digestion, help ease symptoms of lactose intolerance, while also affecting some aspects of metabolism and inflammation, especially when incorporated into a balanced diet (Leeuwendaal et al. 2022; Taylor et al. 2020; Pihelgas et al. 2025). Despite this, studies often fail to clearly differentiate Greek yogurt from other fermented dairy products, with uncertainty persisting around best microbial strains, ideal portion size, as well as direct comparison against other fermented foods (Suvarna and Boby 2005; Chonnacháin et al. 2024).

### 7.1 Dietary Recommendations

There is ample research and information about lactose intolerance, as multiple studies consistently show fermented options like yogurt cause fewer issues compared to non‐fermented milk, since active microbes help break down the sugar. At times, Greek yogurt appears easier on digestion, likely because it holds more protein while containing less lactose, even if direct comparisons remain sparse (Leeuwendaal et al. 2022; Savaiano and Hutkins 2021; Taylor et al. 2020).

Frequent small disruptions in digestion, sometimes paired with constipation, might benefit from consistent intake of Greek‐style yogurt. When considered among broader dietary modifications, this addition can cause more frequent bowel movements and can increase microbe variety within the intestines. Results differ across people, influenced by personal biology as much as ingredient composition. Published observations support these patterns, yet outcomes remain inconsistent (Taylor et al. 2020; Pihelgas et al. 2025).

In groups with elevated risks of heart issues or type 2 diabetes, long‐term observations and controlled experiments repeatedly show that eating yogurt or similar fermented dairy products ties to more stable body weight, healthier blood sugar levels, and fewer cases of metabolic disease along with lower rates of cardiac incidents. Because of these findings, dietary approaches aimed at supporting heart and metabolism often feature plain or low‐sugar Greek yogurt as a sensible choice (Leeuwendaal et al. 2022; Taylor et al. 2020).

Taken together, the physical and clinical evidence reviewed here supports a consistent pattern: Greek yogurt's defined microbial composition produces observable effects on SCFA output, barrier integrity, immune signaling, and metabolic markers, effects that are difficult to credit to more variable fermented foods. Studies show its bacterial strains boost short‐chain fatty acid generation, strengthen gut lining integrity, adjust immunity patterns, and cause favorable shifts in metabolism such as stabilized blood glucose and diminished body‐wide inflammation. Unlike fermented vegetables or other dairy products, this version shows more uniformity across production, an attribute making it particularly reliable for dietary microbiota investigations. Observations from medical trials confirm reduced lactose intolerance symptoms, better stomach and bowel function, alongside added advantages for cardiovascular and metabolic health under consistent intake with a balanced diet (Leeuwendaal et al. 2022; Savaiano and Hutkins 2021; Pihelgas et al. 2025).

### 7.2 Future Directions

Despite these promising findings, significant gaps remain. Future research must separate Greek yogurt from broader dairy categories. Instead of grouping, scientists might specify which starter cultures appear and record physical and chemical traits, including any added microbes, while tracking colony‐forming units (CFU) levels across a product's shelf life (Leeuwendaal et al. 2022; Chonnacháin et al. 2024; Pihelgas et al. 2025). Advanced analytical techniques like immune profiling, metabolomics, and metagenomics can trace shifts in gut microbial communities and link dietary intake to markers tied to inflammation (Chonnacháin et al. 2024; Suvarna and Boby 2005). Trials that directly assess Greek yogurt alongside kefir or fermented vegetables could clarify outcomes linked to dairy‐based fermentation versus those tied to broader categories of fermented items (Wastyk et al. 2021; Leeuwendaal et al. 2022; Suvarna and Boby 2005; Tamang et al. 2016).

Notably, while some rodent studies have looked at generic yogurt or kefir consumption in the context of high‐fat diets or induced colitis, no controlled animal models have directly compared Greek yogurt against other fermented foods using matched observation measures such as SCFA profiles, intestinal lining protein expression, or inflammatory markers. Establishing these comparisons first in mice or rats, where diet, dosage, and baseline microbiota can be tightly controlled, would provide the foundation needed before designing similar human trials. This preclinical work could also clarify whether the higher protein and lower lactose content specific to Greek yogurt independently influence gut microbiome patterns beyond what starter cultures alone influence. Additionally, studies must determine ideal portions, measuring how much Greek yogurt people need to eat, and for how long, to see measurable health benefits, like lower chances of heart disease or colon cancer, or better daily well‐being (Leeuwendaal et al. 2022; Taylor et al. 2020). Finally, research must examine how gut microbes, genetic background, together with lifestyle habits shape reactions to Greek yogurt, allowing for more tailored dietary recommendations (Chonnacháin et al. 2024; Pihelgas et al. 2025).

## Author Contributions

**Jason Dichter:** conceptualization, writing – review and editing, writing – original draft, investigation, visualization.

## Ethics Statement

The author has nothing to report.

## Consent

The author has nothing to report.

## Conflicts of Interest

The author declares no conflicts of interest.

## Data Availability

Data sharing not applicable to this article as no datasets were generated or analysed during the current study.
